# *In Vitro* Evaluation of the Antimicrobial Ability and Cytotoxicity on Two Melanoma Cell Lines of a Benzylamide Derivative of Maslinic Acid

**DOI:** 10.1155/2016/2787623

**Published:** 2016-12-05

**Authors:** Ioana Zinuca Pavel, Corina Danciu, Camelia Oprean, Cristina Adriana Dehelean, Delia Muntean, René Csuk, Danina Mirela Muntean

**Affiliations:** ^1^Department of Pharmacognosy, Faculty of Pharmacy, “Victor Babeş” University of Medicine and Pharmacy, 2 Eftimie Murgu Sq., 300041 Timişoara, Romania; ^2^Department of Pharmaceutical Chemistry, Faculty of Pharmacy, “Victor Babeş” University of Medicine and Pharmacy, 2 Eftimie Murgu Sq., 300041 Timişoara, Romania; ^3^Department of Toxicology, Faculty of Pharmacy, “Victor Babeş” University of Medicine and Pharmacy, 2 Eftimie Murgu Sq., 300041 Timişoara, Romania; ^4^Department of Microbiology and Virology, Faculty of Medicine, “Victor Babeş” University of Medicine and Pharmacy, 2 Eftimie Murgu Sq., 300041 Timişoara, Romania; ^5^Department of Organic Chemistry, Martin-Luther University Halle-Wittenberg, Kurt-Mothes Str. 2, D-06120 Halle (Saale), Germany; ^6^Department of Pathophysiology, Faculty of Medicine, “Victor Babeş” University of Medicine and Pharmacy, 2 Eftimie Murgu Sq., 300041 Timişoara, Romania; ^7^Center for Translational Research and Systems Medicine, “Victor Babeş” University of Medicine and Pharmacy, 2 Eftimie Murgu Sq., 300041 Timişoara, Romania

## Abstract

Maslinic acid is a pentacyclic triterpene extracted from olives that has been systematically reported to exert several therapeutic effects, such as antitumoral, antidiabetic, antioxidant, anti-inflammatory, antiparasitic, and antiviral properties. Recently, new derivatives of maslinic acid have been obtained and expanded the spectrum of biological activities and improved the existing ones. The present study was meant to perform the* in vitro *assessment of the (i) cytotoxic effects of a benzylamide derivative of maslinic acid (“EM2”) (benzyl (2*α*, 3*β*) 2,3-diacetoxy-olean-12-en-28-amide) on B164A5 murine melanoma and A375 human malignant melanoma cell lines and the (ii) antimicrobial activity of the compound on several bacterial strains, respectively. We obtained a dose-dependent cytotoxic effect of EM2 that was particularly relevant to the murine cell line. As on the antibacterial activity, EM2 was tested on 10 bacterial strains* Bacillus cereus, Staphylococcus aureus*,* Streptococcus pyogenes, Streptococcus pneumoniae*,* Enterococcus faecalis*,* Escherichia coli*,* Yersinia enterocolitica*,* Klebsiella pneumoniae*,* Proteus mirabilis*, and* Pseudomonas aeruginosa* and one fungus* Candida albicans*. A significant antimicrobial effect was recorded for* Streptococcus pyogenes* and* Staphylococcus aureus*.

## 1. Introduction

**Figure 1:**
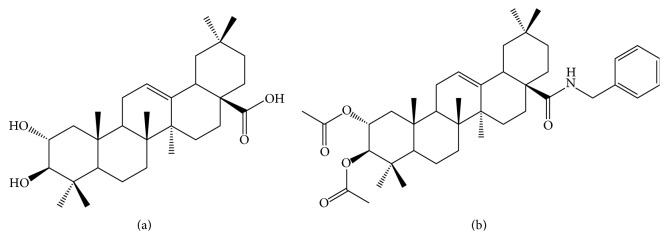
Chemical structure of MA (a) and EM2 (b).

Maslinic acid (MA), a naturally occurring triterpene (Figure 1(a)) mainly obtained from olives (*Olea europaea *L.), has been systematically studied in the past decades for its multiple beneficial effects, including the antioxidant, antitumoral, anti-inflammatory, antidiabetic, cardioprotective, antiparasitic, and antiviral ones [1, 2].

The compound was firstly detected in* Crataegus oxyacantha *L. [1] and further identified in several other plants, vegetables (e.g., spinach and eggplant), and aromatic herbs (e.g., basil) and in fruits (e.g., mandarin, apple peel, and pomegranate) [1, 3]. The group of Gaforio reported the lack of side effects after oral administration in mice, suggesting that MA can be considered a nutraceutical [4].

Derivatization is used to obtain compounds with improved activity, reduced side effects, that will allow to further augment its applicability. Several maslinic acid derivatives have been tested for their proapoptotic property in different cancerous cell lines, including human thyroid, ovarian cancer, lung cancer, colorectal adenocarcinoma, liposarcoma, melanoma, and breast cancer cells [5]. Some of the derivatives displayed increased cytotoxicity and selectivity for tumoral versus nontumoral cells. An acetylated maslinic acid derivative (“EM2”) containing a benzylamide structure has been reported to elicit cytostatic activity and selectivity for tumoral cells while being less cytotoxic for fibroblasts; these observations deserve further investigations [5].

“EM2” (benzyl (2*α*, 3*β*) 2,3-diacetoxy-olean-12-en-28-amide) (Figure 1(b)) used in this study was obtained according to a method consisting in the acetylation of maslinic acid followed by forming the benzylamide, as previously described [5].

Similarly, EM2 has been reported to elicit cytotoxic effect on 518A2 human melanoma cell line with an IC_50_ value of 2 *μ*M, thus being significantly more potent than the parent compound, MA [5]. The group of Antonio Martinez tested several maslinic acid derivatives on B16-F10 murine melanoma cell line and concluded that some of them exhibited a potent effect in terms of apoptosis [6].

Malignant melanoma is one of the most aggressive tumors [7]. In the past decades, the incidence of melanoma has been reported to increase at an alarming rate [8, 9]. While the* in situ* and locally invasive types of melanoma are curable by surgery, the advanced forms are difficult to treat and often fatal [8]. Moreover, malignant melanoma is rapidly evolving tumor with increased metastatic risk [10]. Therefore, adjunct approaches in managing melanoma may include natural compounds, either alone or in combinations.

Beside the rising prevalence of malignant diseases, bacterial resistance to antibiotics represents another worldwide challenge because of the continuous evolution of bacteria with a decrease in developing novel antibacterial agents [11]. An alternative to the classical antibiotics is represented by natural compounds, pentacyclic triterpenes being currently studied [12].

The present study was aimed at characterizing EM2 with respect to its* in vitro* cytotoxic potential in melanoma cells and antimicrobial effect, respectively.

## 2. Materials and Methods

### 2.1. Substances

The chemical structures of maslinic acid (MA) and its derivative (EM2) are presented in Figure 1.

EM2 was prepared as previously reported. In brief, edible, green, pitted olives (6 kg in saline solution) were crushed, dried (110°C, 24 h), and extracted with methanol (5 × 2 L for 4 days each). MA was obtained by chromatography (silica gel, n-hexane/ethyl acetate, 1 : 1) followed by recrystallization (ethyl acetate) (5.65 g, 0.36% of dry mass) [13, 14].

Acetylation of MA (1.2 g, 2.54 mmol) in dry dichloromethane (50 mL) with acetic anhydride (0.96 mL, 10.16 mmol) and NEt_3_ (0.96 mL, 10.16 mmol) for 12 h at 24°C yielded after usual work-up and recrystallization (2*α*, 3*β*) 2,3-diacetoxy-olean-12-en-28-acid (compound** 1**) [15]; 232–234°C [16]. Compound** 1** (200 mg, 0.36 mmol) was dissolved in dry DCM and THF (1 : 1); thionyl chloride and TEA (3 drops) were added at 0°C. After 20 min, the solution was allowed to warm up to room temperature. The solvent was removed under reduced pressure, and the residue was dissolved in DCM and benzylamine (2 mL, 18.4 mmol) and TEA (3 drops) was added. After completion of the reaction (TLC), the mixture was poured into ice water; the precipitate was filtered off, washed with water, and subjected to chromatography (silica gel, n-hexane/ethyl acetate). Benzyl (2*α*, 3*β*) 2,3-diacetoxy-olean-12-en-28-amide (EM2) was obtained after recrystallization from methanol.

DMSO (dimethyl sulfoxide, solvent) was purchased from Sigma-Aldrich.

### 2.2. MTT Cytotoxicity Assay

B164A5 murine melanoma cells and A375 human malignant melanoma cells (Sigma-Aldrich (ECACC and Sigma-Aldrich, origin Japan, stored UK)) were grown in Dulbecco's Modified Eagle's Medium (DMEM; Gibco BRL, Invitrogen, Carlsbad, CA, USA) supplemented with 10% heat-inactivated fetal calf serum (FCS; PromoCell, Heidelberg, Germany) and 1% penicillin/streptomycin mixture (Pen/Strep, 10,000 IU/mL; PromoCell, Heidelberg, Germany) in a humidified atmosphere containing 5% CO_2_, 37°C. Cells were seeded in 96-well microplates at a density of 6000 cells/well and allowed to attach to the bottom of the well overnight. EM2 was added in the following concentrations: 12.5 *μ*M, 25 *μ*M, 50 *μ*M, and 100 *μ*M and incubated for 72 h. Concentration of MTT in the well was as follows: 10 *μ*L of 5 mg/mL MTT (3-(4,5-dimethylthiazol-2-yl)-2,5-diphenyltetrazolium bromide) solution (Sigma-Aldrich) was added in each well (the final volume in the well was 100 *μ*L). The intact mitochondrial reductase converted and precipitated MTT as blue crystals during a 4 h contact period. The precipitated crystals were dissolved in 100 *μ*L of lysis solution provided by the manufacturer (Sigma-Aldrich). Finally, the reduced MTT was spectrophotometrically analyzed at 570 nm, using a microplate reader (Bio-Rad Mark Microplate Spectrophotometer). All* in vitro *experiments were carried out in three microplates with at least five parallel wells. DMSO solvent was used to prepare stock solutions of the tested substances and the highest DMSO concentration (0.3%) of the medium did not have any significant cytotoxic effect on the cells.

### 2.3. Assessment of Antimicrobial and Antifungal Properties

EM2 was tested for its antibacterial activity against 10 bacterial strains* Bacillus cereus *(ATCC 14579)*, Staphylococcus aureus *(ATCC 25923),* Streptococcus pyogenes *(ATCC 19615)*, Streptococcus pneumoniae *(ATCC 49619)* Enterococcus faecalis *(ATCC 29212),* Escherichia coli *(ATCC 25922),* Yersinia enterocolitica *(ATCC 23715),* Klebsiella pneumoniae *(ATCC 700603),* Proteus mirabilis *(ATCC 25933), and* Pseudomonas aeruginosa *(ATCC 27853) and one fungus,* Candida albicans *(ATCC 10231).

The antibacterial activity of this compound was assessed in line with the Guidelines of the National Committee for Clinical Laboratory Standards (NCCLS, 1997) employing the agar disk diffusion method. Bacterial suspensions were adjusted to 0.5 McFarland (with a final bacterial concentration of 1-2 × 10^8^ CFU/mL), except for* Candida albicans*, where the tested suspension was of 2.2 McFarland. The Mueller-Hinton and Sabouraud agar plates were inoculated with suspension using a sterile cotton swab. The stock solutions of the evaluated compound were prepared in DMSO. Sterile Whatman number 1 filter paper disks (6 mm in diameter) impregnated with the solution in DMSO of the test compounds were distributed on the surface of plates inoculated with the bacterial suspension. Then, the plates were incubated at 37°C for 24 h. The inhibition zone diameters were read and reported in millimeters. As control, we used gentamycin (10 *μ*g) for bacilli and staphylococci, gentamycin (120 *μ*g) for streptococci and enterococci and fluconazole (25 *μ*g) for* Candida*. The zone of inhibition for the antibiotics and the antifungal agent was between 16 and 19 mm.

### 2.4. Statistics

For all bacterial strains and fungi, duplicate tests were performed. The results were expressed as mean ± SD. Experiments regarding the cytotoxic activity were carried out in triplicate. The results were expressed as mean ± SEM. Comparison among the groups was performed using the one-way ANOVA test followed by Bonferroni and Dunnett's* post hoc* tests. A *P* value of ≤0.05 was considered to be of statistical significance.

## 3. Results

### 3.1. Cytotoxicity Studies

**Figure 2:**
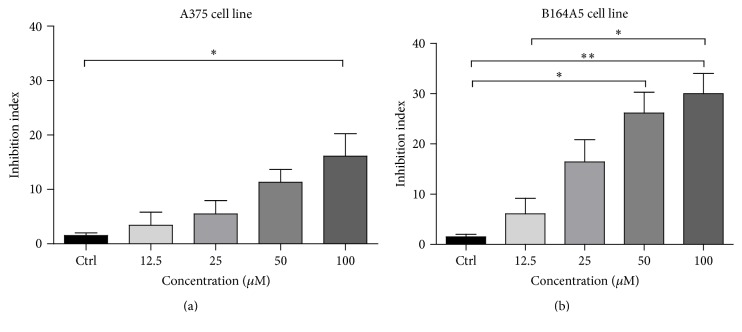
Inhibition index of cells after 72 h of incubation with EM2 in (a) A375 human malignant melanoma cell line and (b) B164A5 murine melanoma cell line.* Data are presented as means ± SEM. The experiments were carried out in triplicate (P* < 0.05 versus* Ctrl)*. *∗* and *∗∗* indicate *P* < 0.05 and *P* < 0.01 as compared with the control group.

As shown in Figure 2, application of EM2 elicited a significant dose-dependent inhibitory effect recorded for both cell lines, B164A5 (Figure 2(b)) and A375 cells (Figure 2(a)). The MTT cytotoxicity assay pointed out an inhibition of growth for A375 and B164A5 cells after EM2 application. In both cell lines, the compound elicited a concentration-dependent cytotoxic effect. The effect was statistically relevant to B164A5 cell line at 50 and 100 *μ*M (26.15 ± 4.15 and 30.00 ± 4.0, respectively, versus Ctrl 1.50 ± 0.50), whereas for A375 cell line only at 100 *μ*M (16.13 ± 4.13 versus Ctrl 1.55 ± 0.42).

The inhibition index was calculated as 1 − absorbance sample *X*/absorbance sample blank.

### 3.2. Studies regarding the Antimicrobial and Antifungal Effects

**Figure 3:**
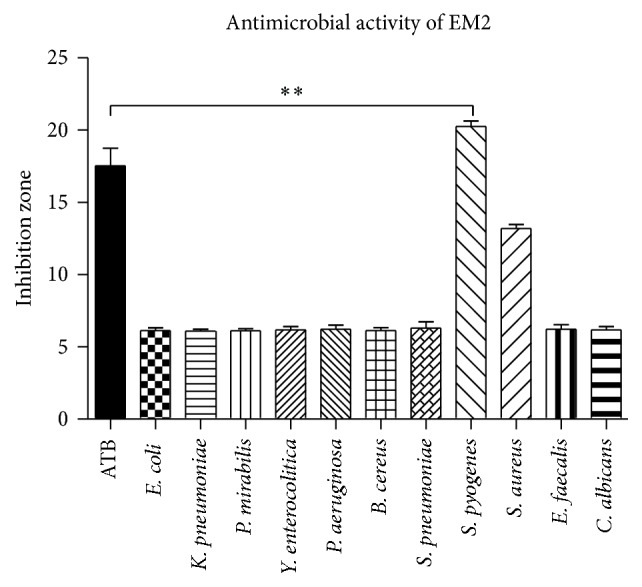
Zones of inhibition (mm) by EM2 for the mentioned bacterial strains and fungus.* Data are presented as means ± SD. The experiments were carried out in duplicate (P* < 0.01 versus* the effects of antibiotics)*. *∗∗* indicate *P* < 0.01 as compared with the control group.

EM2 (10 mM) displayed a significant antimicrobial activity against* Streptococcus pyogenes* (Figure 3). The zone of inhibition for antibiotics and the antifungal agent was between 16 and 19 mm. In the presence of EM2, the inhibition zone for* Streptococcus pyogenes* was 20 ± 0.26 mm, thus suggesting a superior effect of the phytochemical compound as compared to antibiotics. Another bacterial strain sensitive to EM2 was* Staphylococcus aureus* with an inhibition zone of 13 ± 0.19 mm (that, however, did not reach statistical significance). EM2 did not display either antibacterial effects against the other strains presented in Figure 3 or antifungal activity against* Candida albicans*. The concentration used (10 mM) has been chosen according to the results of our dose-titration previous experiments (unpublished data).

## 4. Discussion

The major findings of this paper are that the maslinic acid derivative displayed cytotoxicity against the B164A5 murine melanoma cells and the A375 human malignant melanoma cells, respectively. This observation is in accordance with the results obtained by the group of René Csuk, since the cytotoxic activity of MA and EM2 in 518A2 human melanoma cell line has been previously reported [5]. For 518A2 cells, MA had an IC_50_ value of 13.7 ± 0.9 *μ*M whereas the derivative was very toxic, with an IC_50_ value of 1.5 ± 0.2 *μ*M. When tested on nonmalignant mouse fibroblasts (NiH 3T3 cell line), the IC_50_ was significantly increased, namely, 33.8 ± 3.0 *μ*M. The authors assumed that EM2 is shown to be a promising chemopreventive and chemotherapeutic agent against human melanoma [5]. Furthermore, these authors also showed that EM2 was effective on various other malignant cell lines, namely, A2780, 8505C, A549, HT29, MCF7, DLD1, SW1736, and Lipo (human liposarcoma) [5].

The* in vitro* cytotoxic activity of MA has been systematically explored in several types of cancer, including human colorectal adenocarcinoma (Ht-29 and Caco-2 cell lines) [17, 18], pancreatic cancer (Panc-28 cells) [19], human salivary gland adenoid cystic carcinoma (ACC-2 and ACC-M cell lines) [20], soft tissues sarcoma (SW982 and SK-UT-1) [21], human hepatocellular carcinoma (HepG2 cells) [22], and human breast adenocarcinoma (MCF-7 cell line) [23] (reviewed in [1]). Several maslinic acid derivatives obtained by Parra et al. induced apoptosis in B16F10 murine melanoma cell line. These authors suggested that an increased activity of the derivatives compared to MA is due to the structural changes at C-28 position [6]. Furthermore, it was reported that MA elicited an inhibitory effect in SK-MEL-2 human melanoma cell line proliferation [24].

We acknowledge as a major limitation of this study the use of a single dose of EM2. Accordingly, a possible hormetic behavior that might have been cytotoxic in the human cell line too, even in lower concentrations, cannot be excluded and warrants further investigations.

Regarding the antibacterial effect, we found a significant antimicrobial activity against* Streptococcus pyogenes *and a mild effect against* Staphylococcus aureus, *whereas no activity was observed for* E. faecalis*,* E. coli*,* and P. aeruginosa. *Phytochemicals such as oleanolic and ursolic acid belonging to the same group of pentacyclic triterpenes have been previously reported to exert antibacterial, antiviral, and antiprotozoal effects [25]. Chouaïb et al. evaluated the antimicrobial activity of maslinic and oleanolic acid esters and noted that compounds containing extra sulfur and chlorine substituents were active against* S. aureus, E. faecalis, E. coli*,* and P. aeruginosa *[12]. Kreander et al. tested natural compounds and their synthetic derivatives on erythromycin-resistant bacterial strains of* Streptococcus pyogenes* and* Staphylococcus simulans* and assumed that ursolic acids were active on these bacterial strains [26]. Our data are in line with this latter group's results since EM2 was particularly effective against* Streptococcus pyogenes*.

In the last decades many bacterial strains have developed drug resistance due to bacterial mutations that are difficult to predict; it has been estimated that an increasing number of bacteria will develop resistance to common antibiotics (regardless of their mechanism of action) [11, 27]. Nevertheless, combined, large spectrum antibiotherapy, the solution of choice to overcome bacterial resistance [28], carries the risk of cumulative side effects, in particular ototoxicity (streptomycin) [29], increased incidence of Crohn's disease (tetracycline), cardiovascular arrhythmias, and hearing loss (azithromycin) [30], and so forth.

Research of the 21st century has unveiled a plethora of therapeutic activities for pure natural compounds or total extracts. In this respect, pentacyclic triterpenes are a class of compounds with several valuable effects, including antimicrobial activity [12].

## 5. Conclusion

In summary, the maslinic acid derivative EM2 represents a promising antimicrobial compound, mainly against infections with cocci bacteria. Moreover, the compound also elicited a dose-dependent cytotoxic effect on B164A5 murine melanoma and A375 human melanoma cell lines. The cytotoxic effect of EM2 and the underlying mechanism will be further examined in the* in vivo* experimental models of murine skin cancer.
